# Case report: Repeat coronary function testing in women with ischemia and no obstructive coronary artery disease

**DOI:** 10.3389/fcvm.2023.1096265

**Published:** 2023-07-06

**Authors:** Nissi Suppogu, Benita Tjoe, Janet Wei, Jenna Maughan, Sandy Joung, Odayme Quesada, Chrisandra L. Shufelt, Bruce Samuels, Babak Azarbal, C. Noel Bairey Merz

**Affiliations:** ^1^ Department of Cardiology, University of California, Irvine School of Medicine, Orange, CA, United States; ^2^ Barbra Streisand Women’s Heart Center, Cedars-Sinai Smidt Heart Institute, Los Angeles, CA, United States; ^3^ Women’s Heart Center, The Christ Hospital Heart and Vascular Institute, Cincinnati, OH, United States

**Keywords:** INOCA, women, ischemia, coronary function test (CFT), coronary microvascular dysfunction (CMD)

## Abstract

Women with signs and symptoms of ischemia and no obstructive coronary artery disease (INOCA) often have coronary microvascular dysfunction (CMD). It can be diagnosed by coronary function testing (CFT), which is an invasive coronary angiogram procedure. Frequently, these women have persistent angina despite medical therapy, but it is not clear whether it is due to worsening or persistent CMD or inadequate therapy. In this brief report, we describe findings of repeat CFT in a case series of 12 women undergoing repeat CFT for the assessment of persistent angina in order to better understand the evolving pathology.

## Introduction

More women compared to men have non-obstructive coronary artery disease (CAD); about two-thirds of women with signs and symptoms of ischemia have non-obstructive CAD and often have coronary microvascular dysfunction (CMD) (1). Ischemia with non-obstructive coronary artery disease (INOCA) is not a benign condition as these patients are at increased risk for major adverse cardiovascular events (MACE) including heart failure and myocardial infarction at long-term follow-up (2). CMD can be diagnosed by coronary function testing (CFT), an invasive angiographic procedure using vasoactive substances to assess and delineate pathways of microvascular dysfunction including coronary flow reserve (CFR), a non endothelial dependent pathway, and coronary blood flow (CBF), an endothelial independent microvascular pathway (3). Medical therapy targeted towards abnormal CFT pathways has been shown to improve angina and quality of life at 1-year follow-up (4). We hypothesized that repeat CFT may help better our understanding of pathophysiological progression of abnormal pathways in CMD while the patients are on therapeutics and have ongoing symptoms. This may further help with management options. In this brief retrospective case report, we studied 12 women who were diagnosed initially with invasive CFT and found to have CMD who then underwent clinically ordered repeat testing for further evaluation of persistent angina despite medical management, with subsequent pathway findings and treatment outcomes.

## Methods

Among 280 women and men at a single tertiary center enrolled in a registry, 12 (4.3%) women underwent repeat CFT per previously published methods in the women's ischemia syndrome evaluation (WISE) studies (3, 5). Long-acting calcium channel blockers (CCB) were held for 48 h while short-acting CCB, angiotensin-converting enzyme inhibitors (ACEi), angiotensin receptor blockers (ARBs), beta blockers (1), and long-acting nitrates were held for 24 h and sublingual nitroglycerin for 4 h per-procedurally. Non-obstructive CAD (<50% stenosis) was confirmed and function testing was then performed using a Doppler FloWire (Philips Volcano) in the left anterior descending artery (3). Non-endothelial dependent microvascular dysfunction was defined as a coronary flow reserve (CFR) ≤2.32 to graded doses of 18 mg and 100 mg of intracoronary (IC) adenosine, a threshold associated with elevated risk of MACE as seen in several WISE studies (6, 7). Endothelial dependent microvascular dysfunction was defined as ≤50% increase in coronary blood flow (CBF) to IC acetylcholine (ACH). Both abnormal CFR and abnormal CBF have been shown to have adverse outcomes with elevated risk of MACE (7). Endothelial dependent macrovascular dysfunction was defined as ≤0% increase in coronary artery diameter to 36.4 mcg of IC ACH. Epicardial vasospasm was defined as >75% vasoconstriction to ACH compared to coronary artery diameter post-nitroglycerin, with chest pain and ECG changes. Non-endothelial dependent macrovascular dysfunction was defined as <20% increase in coronary diameter to IC nitroglycerin (3). Angina was assessed using the Seattle Angina Questionnaire (SAQ) following the initial and repeat CFT. Statistical tests were paired *t* tests. A significance level of 0.05 was used for all tests.

### Patient characteristics and outcomes

**Figure 1:**
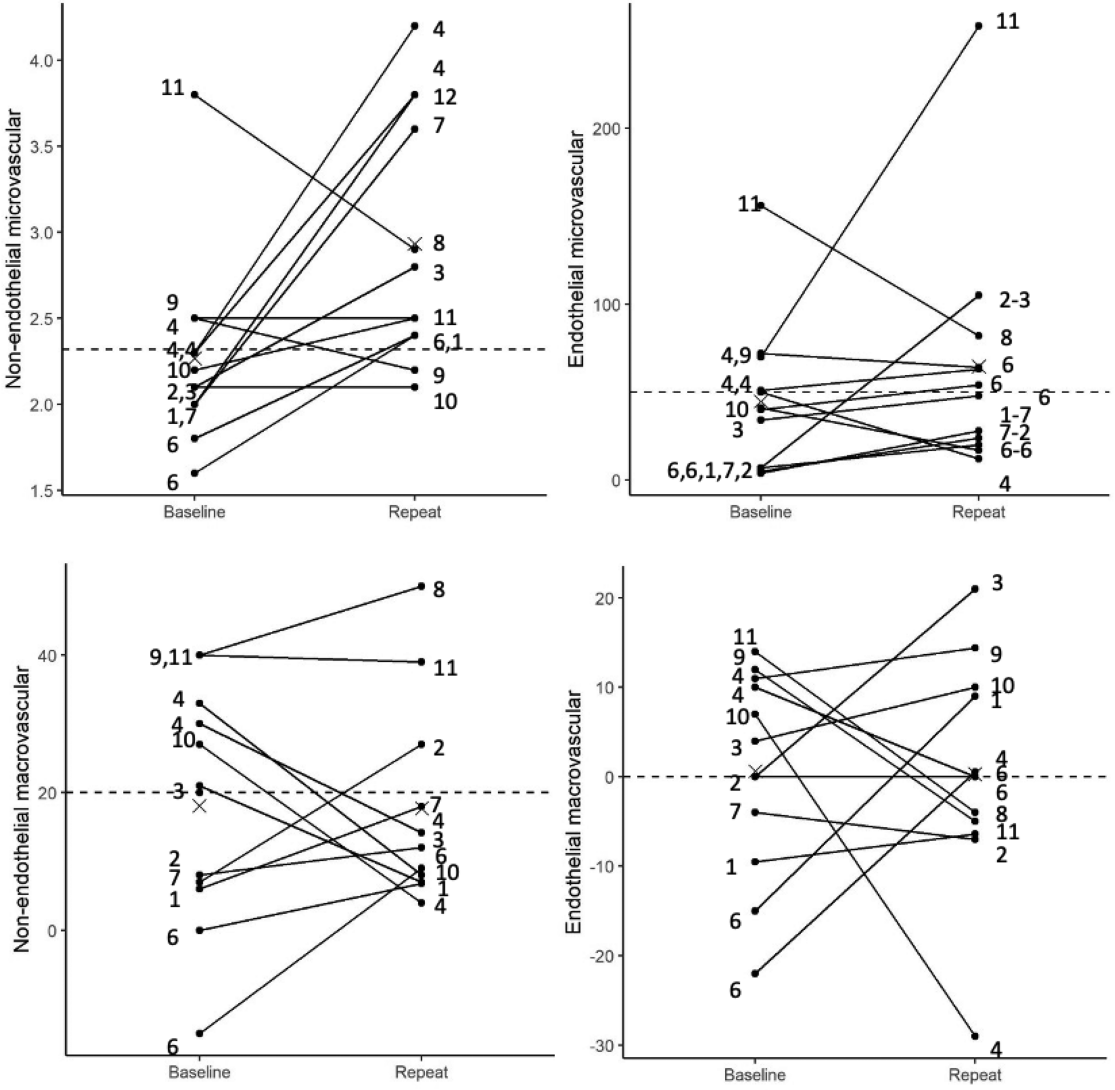
Interaction plot showing baseline CFT pathways and their end points at repeat over a period of time. Numbers on the left of the individual plots indicate combination of medication's patients were on before the initial CFT and numbers on the right of the plot indicate the combination of medication patients were on at the time of the repeat CFT. 1, aspirin and statins; 2, aspirin, statins, calcium channel blockers; 3, aspirin, statins, calcium channel blockers and nitro; 4, aspirin, statin, betablocker, nitro; 5, aspirin, statins, betablockers, nitro, ranexa; 6, statins; 7, statin, betablockers, calcium channel blockers; 8, statins, betablocker, ranexa; 9, statins, betablocker, nitro, ranexa; 10, statin, calcium channel blockers and nitro; 11, betablocker, nitro and ranexa.

At baseline, mean group age was 52 ± 10.5 years with 75% post-menopausal women, all were nonsmokers, 17% diabetic, 25% hypertension and 25% hyperlipidemia. Median time between initial CFT and follow-up CFT was 8 years (range 2–11). At repeat CFT, all patients remained with non-obstructive atherosclerosis and no new diagnosis of diabetes, hypertension, or smoking. Findings of initial and repeat micro- and macrovascular CMD pathways are shown (Table 1). At baseline CFT, 92% of patients had abnormal CFR ≤ 2.32 compared to 50% at follow-up, with a significant overall improvement (2.27 ± 0.55–2.93 ± 0.72, respectively; *p* = 0.03) (Figure 1). Increase in CBF was 41.9% ± 44.2% at baseline compared to 64.6% ± 67.2% at repeat testing (*p* = 0.33). Other pathways did not show a significant improvement (Figure 1). Two patients were found to have epicardial vasospasm on the repeat CFT that was not observed on initial testing. Heart rate and blood pressure readings were not significantly different at the time of initial and repeat CFT as shown (Table 2).

**Table 1:** Coronary microvascular dysfunction (CMD) pathways at baseline and repeat coronary function testing (CFT).

Patient	Age at Baseline CFT	Years between Baseline and Repeat CFTs	Coronary Microvascular Dysfunction Pathways							
			Microvascular				Macrovascular			
			Non-endothelial^a^		Endothelial^b^		Endothelial^c^		Non-Endothelial^d^	
			Baseline	Repeat	Baseline	Repeat	Baseline	Repeat	Baseline	Repeat
1	65	9	3.8	2.9	51	63	**−9.5**	**−6.4**	**0**	**6.8**
2	53	11	2.5	2.5	**5**	**24**	11	14.4	**7**	27
3	43	2	**1**.**6**	2.4	**7**	105	**0**	**0**	30	**14.2**
4	52	2	**2**.**3**	3.8	**50**	**12**	10	**0**	40	50
5	47	2	2.5	**2**.**2**	**34**	**48**	7	**−29**	**20**	**0**
6	56	8	**2**	3.6	**40**	54	**−4**	**−7**	21	**7**
7	40	9	**2**.**3**	4.2	70	258	**−22%**	0.55	**6**	**18**
8	44	8	**2**.**1**	2.8	**41**	**17**	14	**−4**	27	**4**
9	58	6	**2**	3.8	7	**20**	4	10	**8**	**12**
10	52	4	**2**.**2**	2.5	156	82	12	**−5**	33	**8**
11	30	9	**1**.**8**	2.4	72	64	**0**	21	40	39
12	48	9	**2**.**1**	**2**.**1**	**4**	**28**	**−15**	9	**−15**	**9**

^a^ Non-endothelial dependent microvascular dysfunction: CFR ≤ 2.32.

^b^ Endothelial dependent microvascular dysfunction: ≤50% increase in coronary blood flow in response to acetylcholine.

^c^ Endothelial dependent macrovascular dysfunction: ≤0% increase in coronary artery diameter in response to acetylcholine.

^d^ Non-endothelial dependent macrovascular dysfunction: ≤20% increase in coronary artery diameter in response to nitroglycerin.

Bolded indicates abnormal.

**Table 2:** Herat rate and blood pressure (systolic and diastolic) at the time of initial and repeat coronary function testing.

	Heart rate bmp			Systolic Blood Pressure mmHg			Diastolic Blood Pressure mmHg		
Patient	Baseline CFT	Repeat CFT	HR change	Baseline CFT	Repeat CFT	SBP change	Baseline CFT	Repeat CFT	DBP change
1	58	54	−4	125	104	−21	73	62	−11
2	65	62	−3	99	101	2	67	57	−10
3	57	65	8	189	117	−72	81	64	−17
4	75	75	0	119	128	9	77	83	6
5	85	91	6	111	124	13	55	74	19
6	80	77	−3	136	131	−5	75	76	1
7	91	60	−31	131	100	−31	84	62	−22
8	57	70	13	131	133	2	74	89	15
9	56	63	7	123	109	−14	63	82	19
10	71	78	7	123	108	−15	67	64	−3
11	87	85	−2	98	99	1	72	68	−4
12	84	90	6	126	102	−24	86	70	−16

**Figure 2:**
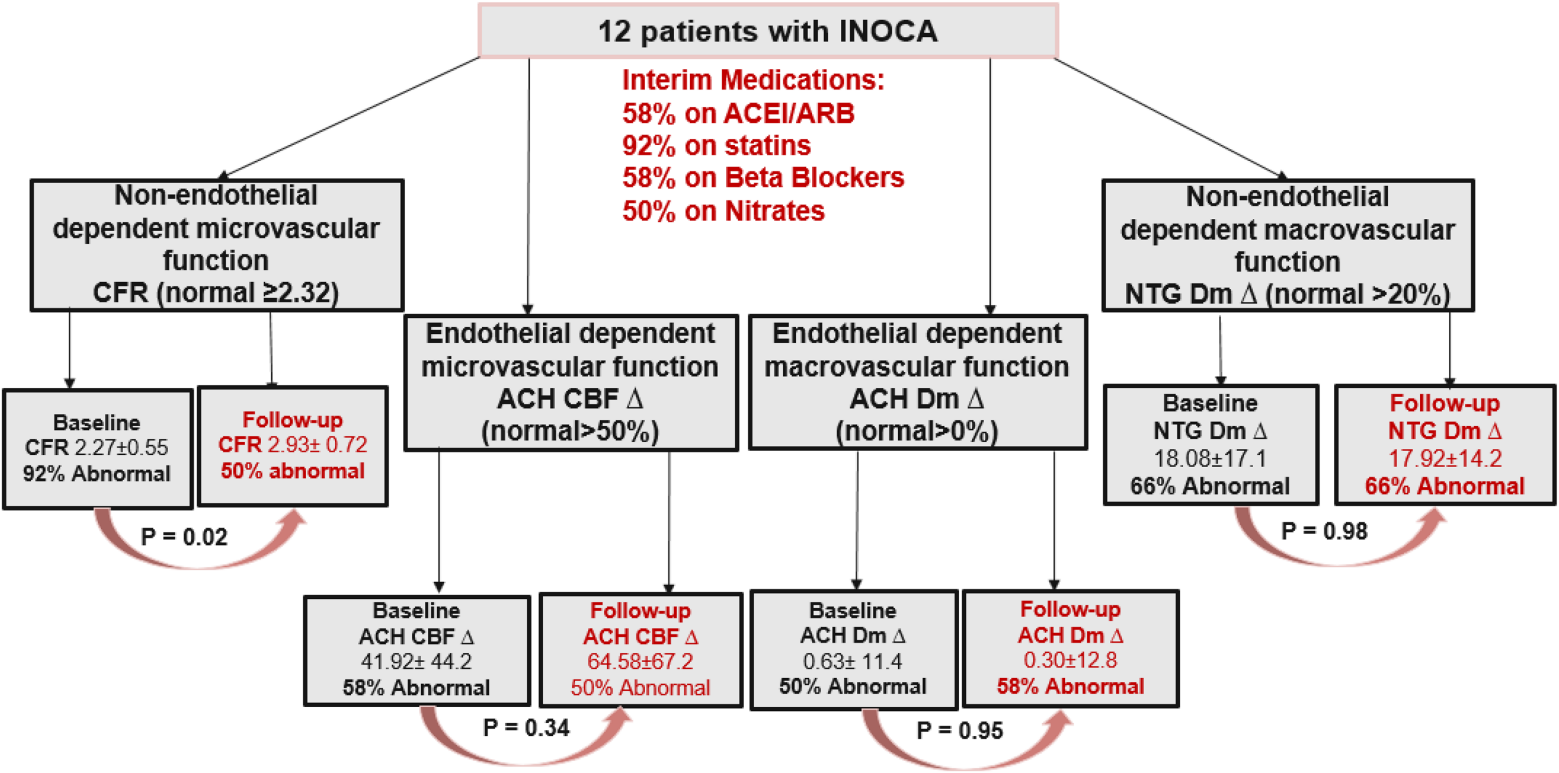
Flow chart of the case series identifying CMD pathway at baseline and at repeat CFT along with the significant changes in percentages in each of the pathways involved. INOCA, ischemia with non-obstructive coronary artery disease; CFR, coronary flow reserve; ACH, acetylcholine; CBF, coronary blood flow; Dm, diameter; NTG, nitroglycerin; ACEi, angiotensin converting enzyme inhibitor; ARB, angiotensin II receptor blocker.

Following the initial CFT, 58% of patients were initiated on ACEi/ARBs or BB, while 50% were initiated on nitrates**.** At the time of repeat CFT, 92% of the twelve patients were being treated with a statin, 92% with an antiplatelet agent, 58% with BB, 58% with nitrates, 50% with ACEi/ARB, 33% with CCB and 25% with ranolazine. All patients with improved CFR were either on statin/BB or ACEi prior to repeat CFT (Table 3) (Figure 2).

**Table 3:** Medications at the time of repeat coronary function testing (CFT).

Patient	Years between Initial and Repeat CFT	Medications						
		ACEi/ARB	Statin	BB	CCB	Nitrates	Ranexa	Antiplatelets
1	9	0	1	0	0	0	0	1
2	11	1	1	0	0	0	0	1
3	2	0	1	1	1	0	0	1
4	2	1	1	0	1	0	0	1
5	2	1	1	0	1	1	0	1
6	8	0	1	0	1	1	0	1
7	9	1	1	1	0	1	0	1
8	8	1	1	1	0	1	0	1
9	6	1	1	1	0	1	0	1
10	4	0	1	1	0	1	1	1
11	9	0	0	1	0	1	1	0
12	9	0	1	1	0	0	1	1

0, not taking medication; 1, taking medication.

ACEi, angiotensin converting enzyme inhibitor; ARB, angiotensin II receptor blocker; BB, beta blocker; CCB, calcium channel blocker.

At baseline CFT, the mean SAQ score was 40.1 ± 9.5. It showed a trend towards improvement at repeat to 53.5 ± 3.10 (*p* = 0.08). Amongst the SAQ domains, there was a trend toward improvement in treatment satisfaction from 69 ± 17.6 to 84 ± 13.5 (*p* = 0.096). There was also a significant improvement in disease perception from 33 ± 17.8 to 50 ± 24.2 (*p* = 0.001). There was no significant improvement in angina stability (*p* = 0.71), angina frequency (*p* = 0.28), and physical limitation (*p* = 0.57) scores.

## Discussion

To our knowledge, this is the first case report examining repeat coronary function testing for reassessment of CMD in women with persistent angina. Overall, there was significant improvement in CFR on repeat testing (*p* = 0.02) that is noteworthy in the context of studies demonstrating a worsening of CFR with age. Notably, this cohort of women reported persistent symptoms (8). In many patients, CFT revealed persistent abnormalities in both macro- and microvascular endothelial function and newly diagnosed epicardial vasospasm spasm in 2 of the 12 cases that may be the culprit of ongoing angina.

Targeted medical management of CMD aims to treat the underlying pathophysiology and pathway of dysfunction (2). Statins have been shown to have both anti-atherosclerotic and anti-inflammatory properties and improve CFR (9). A greater atherosclerotic burden has been observed in patients with CMD, progressing and possibly leading to obstructive CAD over 10 years (10). It can be seen that the majority of our patients (92%) were on statins and had minimal progression of atherosclerosis and also an improvement in CFR.

ACEi therapy for CMD patients is associated with improved symptoms and outcomes, likely driven by improved endothelial function and microvascular tone (11). Only 50% of our patients were on ACEi/ARBs at follow-up, as they had either side effects or low blood pressure limiting therapy, and there was not a significant improvement in CBF at follow-up. CCBs have shown to improve microvascular tone and epicardial vasospasm (11). Ranolazine has been shown to improve angina in women with low CFR (12). For patients with abnormal nociception, TCAs may improve angina likely via visceral analgesic effects (11).

Repeat CFT findings helped guide therapeutic alterations to specified abnormal pathways in a few patients in this small case series. In patients with more observed atherosclerosis, the statin dose was increased. In those with persistent constrictive abnormalities consistent with endothelial dysfunction, ACEi/ARBs were preferentially initiated. In patients with newly observed vasospasm, dual CCB therapy and as needed nitrates were favored for vasodilation. Repeat CFT in our case series also helped in delineation of pathways over a period of time, which is particularly useful in patients with physiological limitations and intolerance to existing therapies. For example, it helped us look further into patients with low blood pressure that may limit anti-hypertensive therapy and help utilize other drug classes. At any given time, the patients with improvement in CFR were either on statins, beta blockers, or ACEi, possibly influencing the improvement in microvascular function in a positive way.

The observed improvement in overall SAQ and significant improvement in treatment satisfaction highlights also that targeted management informed by invasive CFT findings can improve patient reported outcomes. This is consistent with findings from the randomized controlled CorMicA trial which demonstrated that stratified therapy as guided by CFT improves angina and quality of life, compared to standard care following a sham invasive diagnostic procedure (4). It was also evident in our case analyses that many of these patients underwent several non-invasive stress tests both prior to initial CFT and at times between the repeated CFTs. There are different modalities of testing, such as PET, cardiac MRI, and echo doppler, that can diagnose CMD; however, these imaging modalities are surrogates of flow only and need special expertise and equipment as well (13). While these tests can provide useful information regarding ischemia and functional capacity, repeated testing may warrant unnecessary healthcare costs and yield limited findings to guide management.

This is a small retrospective analysis of 12 patients with CMD and further limited to women. Protocol change was seen from initial to repeat testing at times—several women did not participate in vasospasm studies (high dose ACH) during the initial study, and some were not given a higher dose of adenosine per the study design. Limited data are available on the reproducibility of CFR, though randomized trials have demonstrated minimal variability in placebo control groups as compared to ACEi (14). Lastly, there exists intrinsic bias in patient selection, as those undergoing repeat CFT were those in our clinic presenting with persistent angina and lack of improvement or intolerances to medical therapy. It is possible that patients with improvement in symptoms could show improvement in other pathways not observed in this analysis. It is also possible that a placebo effect of guided treatment may affect self-reported outcomes.

## Conclusions

This brief report demonstrates that CMD is possibly a persistent condition and that targeted interventions guided by invasive testing may be associated with improvement in CFR and patient-reported treatment satisfaction in follow-up. Clinical trials are underway (15) to better understand the impact of the anti-anginal and anti-ischemic medications on CMD pathways in order to tailor treatment guidelines for CMD in the setting of INOCA.

## Data Availability

The raw data supporting the conclusions of this article will be made available by the authors, without undue reservation.
